# Validating Serum S100B and Neuron-Specific Enolase as Biomarkers for the Human Brain – A Combined Serum, Gene Expression and MRI Study

**DOI:** 10.1371/journal.pone.0043284

**Published:** 2012-08-14

**Authors:** Daniel-Paolo Streitbürger, Katrin Arelin, Jürgen Kratzsch, Joachim Thiery, Johann Steiner, Arno Villringer, Karsten Mueller, Matthias L. Schroeter

**Affiliations:** 1 Max Planck Institute for Human Cognitive and Brain Sciences, Leipzig, Germany; 2 Clinic for Cognitive Neurology, University of Leipzig, Leipzig, Germany; 3 Leipzig Research Center for Civilization Diseases, University of Leipzig, Leipzig, Germany; 4 Institute of Laboratory Medicine, Clinical Chemistry and Molecular Diagnostics, University of Leipzig, Leipzig, Germany; 5 Department of Psychiatry, University of Magdeburg, Magdeburg, Germany; 6 Pembroke College, University of Cambridge, Cambridge, United Kingdom; 7 Consortium for Frontotemporal Lobar Degeneration, Ulm, Germany; Centre Hospitalier Universitaire Vaudois Lausanne - CHUV, UNIL, Switzerland

## Abstract

**Introduction:**

Former studies have investigated the potential of serum biomarkers for diseases affecting the human brain. In particular the glial protein S100B, a neuro- and gliotrophin inducing plasticity, seems to be involved in the pathogenesis and treatment of psychiatric diseases such as major depression and schizophrenia. Neuron-specific enolase (NSE) is a specific serum marker for neuronal damage. However, the specificity of these biomarkers for cell type and brain region has not been investigated in vivo until now.

**Methods:**

We acquired two magnetic resonance imaging parameters sensitive to changes in gray and white matter (T_1_-weighted/diffusion tensor imaging) and obtained serum S100B and NSE levels of 41 healthy subjects. Additionally, we analyzed whole brain gene expressions of S100B in another male cohort of three subjects using the Allen Brain Atlas. Furthermore, a female post mortal brain was investigated using double immunofluorescence labelling with oligodendrocyte markers.

**Results:**

We show that S100B is specifically related to white matter structures, namely the corpus callosum, anterior forceps and superior longitudinal fasciculus in female subjects. This effect was observed in fractional anisotropy and radial diffusivity – the latest an indicator of myelin changes. Histological data confirmed a co-localization of S100B with oligodendrocyte markers in the human corpus callosum. S100B was most abundantly expressed in the corpus callosum according to the whole genome Allen Human Brain Atlas. In addition, NSE was related to gray matter structures, namely the amygdala. This effect was detected across sexes.

**Conclusion:**

Our data demonstrates a very high S100B expression in white matter tracts, in particular in human corpus callosum. Our study is the first in vivo study validating the specificity of the glial marker S100B for the human brain, and supporting the assumption that radial diffusivity represents a myelin marker. Our results open a new perspective for future studies investigating major neuropsychiatric disorders.

## Introduction

Former studies investigated the potential of serum proteins as biomarkers for brain diseases. In particular, the glial protein S100B has been discussed in this context [1]–[3]. S100 proteins influence various cellular responses along the calcium-signal-transduction pathway [2], [4], [5]. S100B is localized in and may be secreted by astro- and oligodendrocytes. In nanomolar concentrations it acts as growth and/or differentiation factor for neurons and glia, whereas in micromolar levels S100B may have deleterious effects and induces apoptosis due to an increased expression of proinflammatory cytokines [6], [7]. Recently, it has been suggested that S100B may play a crucial role in the pathogenesis and treatment of frequent psychiatric disorders such as major depression and schizophrenia [1], [7]–[13]. Antidepressive drugs lead via the serotonergic system to astrocytic S100B secretion, which in turn may induce neurogenesis required for the behavioral effects of antidepressants [8], [14]. Consequently, S100B-related mechanisms have been proposed as potential targets for novel antidepressive therapies.

Accordingly, one might assume that increased serum S100B might reflect an ongoing regenerative action in neuropsychiatric diseases. However, serum S100B, which might not reflect local concentrations in the brain, was rather elevated in acute stages of depression and schizophrenia and positively correlated with depressive symptoms and negative symptoms in schizophrenia. Accordingly, clinical data suggest serum S100B rather as a pathological biomarker than plasticity marker [7]–[9]. In agreement with these arguments genetic studies demonstrated that S100B is a susceptibility gene for mood disorders and schizophrenia [1], developmental dyslexia [3] and cognitive dysfunction [15]. In sum, serum S100B may constitute a diagnostic, prognostic and treatment biomarker for major depression, schizophrenia and neurological diseases [16], [17]. Beside S100B, neuron-specific enolase (NSE) has been suggested to be a specific serum marker for neuronal damage. Because NSE is primarily localized in the cytoplasm of neurons [18]–[22] and is not secreted, increases in cerebrospinal fluid or blood indicate structural damage to neuronal cells.

Although serum S100B and NSE offer the opportunity to easily investigate the function of or damage to glial and neuronal structures in neuropsychiatric disorders [8], [23], [24], the specificity of these biomarkers for cell type and brain region have not been investigated in vivo until now. Magnetic resonance imaging (MRI) is very sensitive in identifying regional structural changes in the human brain. Voxel based morphometry (VBM) [25] has enabled a wide variety of studies focusing on gray matter (GM) changes [26]–[28] in various diseases [29]–[32], due to training [33]–[35] or genetic effects [36]–[38] using T_1_-weighted images. With another MRI-based approach, diffusion tensor imaging (DTI) [39], one may specifically analyze white matter (WM) changes, for instance with the software package tract-based spatial statistics (TBSS) developed by the Oxford Centre for Functional MRI of the Brain (FMRIB) [40]. In addition to the most important DTI parameter fractional anisotropy (FA) [41] indicating global WM integrity [42], axial and radial diffusivity are markers for axonal and myelin degeneration [43].

Our study aimed to isolate the regional neural correlates of glial (S100B) and neuronal (NSE) serum markers with two MRI parameters sensitive to changes in the GM and WM of the human brain. We hypothesized that S100B as an astro- and oligodendrocytic marker is associated with GM and WM parameters. For the WM, we expected the strongest effect in the corpus callosum, because this structure has an abundance of oligodendrocytes according to histological studies [6] and shows the highest signal-to-noise ratio due to parallel orientation of numerous fibers [44]. Additionally, we hypothesized that NSE as a neuronal marker is associated with GM parameters.

To avoid any influences of diseases on the association between regional MRI markers and serum S100B and NSE, we involved only healthy subjects. It is well known that S100B is age [45]–[47], sex [46], [48] and weight dependent [49]–[51], while influences of age or sex on NSE are controversially discussed [52]–[54]. Moreover, recent studies combining serum markers and WM parameters found different results for female and male subjects [44]. To control for these influences, we analyzed data separately in female and male subjects, included a wide age range, and controlled for influences of age, body mass index (BMI) and total intracranial volume (TIV). Finally, we validated our results by additionally investigating gene expression in the whole human brain genome wide atlas of the Allen Institute for Brain Sciences and by histological co-localization studies in human post mortem brain and in cell culture.

## Methods

### Subjects

Forty-one healthy Caucasian adults participated in the study (20 male/21 female; mean age 50.35±21.61/46.38±24.44 years; range 21–74/20–79). All participants gave written informed consent. They completed a health history questionnaire. The research protocol was approved by the Ethics Committee of the University of Leipzig, and was in accordance with the latest version of the Declaration of Helsinki.

### T1-weighted Magnetic Resonance Imaging

Scanning was performed on a 3T TIM Trio device (Siemens Medical Solutions, Erlangen, Germany). *T*_1_-weighted images were acquired with a three-dimensional magnetization prepared rapid gradient echo (MP-RAGE) sequence using the following parameters: inversion time 650 ms, repetition time *(TR)* = 1.3 s, *TR* of the gradient-echo kernel 10 ms, echo time 3.46 ms, flip angle 10°, bandwidth 130 Hz/pixel, acquisition matrix 256×240, field of view 256×240 mm^2^, slab thickness 192 mm (sagittal orientation), 128 partitions, 95% slice resolution. Reconstructed images were obtained with a nominal voxel size of 1×1×1 mm^3^.

### Diffusion Tensor Imaging

Diffusion-weighted images were acquired with twice-refocused spin echo echo-planar-imaging sequence [46], TE = 100 ms, TR = 12 s, 128×128 image matrix, FOV = 220×220 mm^2^: 88 axial slices (no gap); voxel size: 1.72×1.72×1.7 mm^3^. Additionally, fat saturation was employed together with 6/8 partial Fourier imaging and generalized auto-calibrating partially parallel acquisitions (GRAPPA [55], acceleration factor  = 2). Diffusion weighting was isotropically distributed along 60 diffusion-encoding gradient directions with a b-value of 1000 s/mm^2^.

### Measurement of Serum Markers

Blood samples were obtained by venipuncture from subjects. Analysis was performed as previously described in detail [8], [9]. S100B and NSE were measured by monoclonal 2-site immunoluminometric assays performed on the fully mechanized system LIAISON (Diasorin, Dietzenbach, Germany). The detection limit for the assays was 0.02 µg/l and 0.04 µg/l.

### Voxel Based Morphometry

T1-weighted images were processed using VBM8, implemented by Gaser *et al.* (http://dbm.neuro.uni-jena.de/vbm/) for the Statistical Parametrical Mapping software (SPM, ver. 8.xx). GM segments were non-linearly registered, modulated [56] and smoothed using a 5 mm^3^ smoothing-kernel. Resulting images were fed into a multiple regression model using S100B (or NSE) as regressor of interest and NSE (or S100B), BMI, TIV and age as confounding covariates. GM images were threshold to exclude voxels with a GM probability below 10% prior to the statistical analyses. Significant resulting brain structures were identified by the TD-ICBM Human Atlas [57] and a volume of interest (VOI) analysis over both groups of subjects was created based on this masks. Averaged voxel values were correlated with the obtained biomarker levels using partial correlation in MATLAB (version 7.11.0/R2010b, Natick, Massachusetts: The MathWorks Inc., 2012) controlling for the same parameters as described above. To proof the significance of the difference of the correlation coefficients we used a formula which has been described in [58].

### Tract-Based Spatial Statistics

Secondly, we analyzed DTI datasets using TBSS from the FMRIB Software Library (FSL) [40]. We followed the recommended preprocessing steps with default parameters described on their homepage (http://www.fmrib.ox.ac.uk/fsl/tbss/index.html). After preprocessing, we fed the images into a multiple regression analysis using FSLs ‘randomise’ program, with the same statistical design as it is described above and computed a randomize statistics with 100.000 permutations. Results were thresholded by the threshold-free cluster enhancement (TFCE) approach (*p*<0.05 corrected). TFCE is a technique which aims to enhance cluster-like structures and simultaneously ignores noisy regions in statistical images without explicitly defining a hard-threshold-based clustering [59]. Furthermore, we followed the same VOI statistical procedure for significant identified brain structures as above. Due to the different analyzed tissue we used the anatomical atlas from TD-ICBM Human Atlas to the Johns Hopkins University ICBM white-matter-labels [60]. All statistics, TBSS as well as VOI analysis, were applied on the skeletonized FA, radial (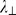) and axial diffusivity data (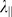).

### Histological Analyses – Post Mortem and Cell Culture

The brain of a 32 year old Caucasian woman (who died from acute myocarditis associated with lupus without brain involvement), which is not included in our imaging study, was removed from the cranium within 20 hrs after death. A tissue sample from the corpus callosum was fixed in 4% buffered paraformaldehyde (pH 7.4), cryoprotected, and rapidly frozen at −20°C using 2-methylbutane. Serial sagittal 20 µm thick sections were cut on a cryostat (Jung Frigocut 2800 E, Leica, Bensheim, Germany). Oligodendroglial OLN-93 cells were obtained from the Richter-Landsberg lab (Oldenburg, Germany) and cultured as previously described [11]. Free-floating sections or OLN-93 cultures were washed and incubated with the corresponding antibodies: (i) polyclonal rabbit anti-recombinant-S100B 1∶50; (ii) monoclonal mouse anti-myelin basic protein 1∶100, (iii) monoclonal mouse anti-p75 neurotrophin receptor 1∶100 with 0.3% Triton X-100 and 1% normal goat serum overnight at 4°C. This step was followed by incubation for 3 h with the respective secondary antibodies at a 1∶500 dilution: Alexa Fluor 546 (goat anti-rabbit-IgG; red fluorescence) and Alexa 488 (goat antimouse-IgG; green fluorescence). Specimens were examined using a fluorescence microscope (Axiophot; Zeiss, Jena, Germany) equipped with phasecontrast, fluorescein and rhodamine optics. The specificity of the immunoreactions was controlled by the application of buffer instead of the primary antiserum.

### Whole Brain Genome Expression Analysis

The Allen Human Brain Atlas is a publicly available online resource of gene expression (www.human.brain-map.org; [61]). It characterizes gene expression in human brain tissue with genome-wide microarray-based gene expression profiles including over 62,000 gene probes for 500 samples from each hemisphere covering the whole brain. Tissue samples collected for microarray analysis were processed for RNA isolation, quantification, and normalization. Microarray analysis data, normalized across each brain, are included in the Allen Human Brain Atlas dataset and illustrated in heat map format as z scores. Z scores represent individual regional gene expression normalized to whole brain expression of that gene. When there are multiple samples for a given structure and subject, values in the heat map are average values. Quality control was performed several times. To date, three subjects without a history of neuropsychiatric or neurological conditions are contained in the database (H0351.1009, H0351.2001, H0351.2002; age 57, 24, 39 years; all male; ethnicity white/Caucasian [.1009], and black/African American [.2001/.2002]). Note that these subjects were not included in our imaging study. Detailed information for subjects included and analysis methods is available at www.human.brain-map.org. To calculate mean values of normalized expression in brain regions of interest we extracted normalized z scores from the database for each individual and region of interest. Thereafter, expression values were compared with two-tailed Student’s t tests.

## Results

### Serum Markers

Because serum S100B might be influenced by sex, age and body mass index, we controlled for these factors. In accordance with literature data [48] mean serum S100B levels were higher in female (65.7±26.6 ng/l) compared to male subjects (54.0±16.4 ng/l; ‘trend’ *p* = 0.1), whereas there were no sex related differences for serum NSE (females 9.68±1.75 µg/l; males 10.27±1.96 µg/l; *p* = 0.31; two-sample Student’s t-test; generally, *p* values for two tailed tests). Serum S100B and NSE values did not correlate within the group of female subjects (*p* = 0.22), but correlated positively within the group of male subjects (*p*<0.05; Pearson’s correlation). Age and body mass index were not correlated with S100B or NSE if calculated in female and male subjects alone or in the whole group. Accordingly, we analyzed correlations between serum markers as regressors of interest and imaging parameters in both sex groups separately and, in general, controlled for the respective second serum marker beside age and body mass index (and total intracranial volume) as confounding covariates in the following analyses.

### Combining Serum Markers and Diffusion Tensor Imaging

Both S100B and NSE serum markers were analyzed for their explanatory power for the DTI data, separately for FA, radial and axial diffusivity. For NSE, we did not obtain any significant results with the chosen statistical threshold. In contrast, serum levels of S100B were correlated with regional DTI parameters in female subjects, which was not the case for male.

**Figure 1:**
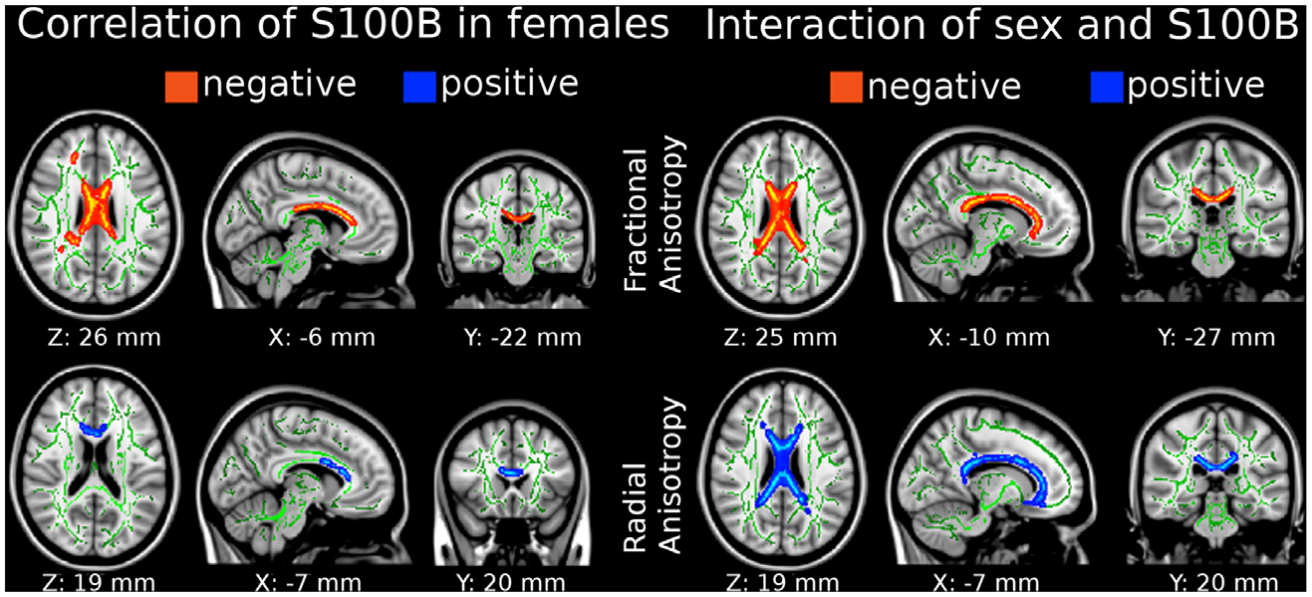
Diffusion tensor imaging parameters correlate with serum S100B mainly in the corpus callosum, anterior forceps, and the right superior longitudinal fasciculus of the female brain (left) in comparison with male brains (right). Clusters are corrected for multiple comparisons (*p*<0.05, threshold-free cluster enhancement), positions are reported in MNI-coordinates. There was no significant correlation of S100B in male brains alone using multiple comparison correction and very little using uncorrected thresholds (not shown). Radiological convention (left side of the brain is presented on the right).

TBSS results are presented in Figure 1. We show a significant negative correlation between S100B and FA across the anterior portion (rostrum, genu) and body of the corpus callosum, particularly the right anterior forceps, and the right superior longitudinal fasciculus (left upper row of Figure 1). No significant effects were detected for the posterior portions of the corpus callosum (splenium, tapetum). Additionally, we were able to show a significant positive correlation of S100B with radial diffusivity but not with axial diffusivity for the genu and anterior body of the corpus callosum, and for the anterior forceps (left lower row of Figure 1). In male subjects no such an association was detected – applying both corrected and uncorrected thresholds.

We aimed to confirm this result and compare female and male groups in a regional analysis focusing on the most consistent brain region, the corpus callosum. As illustrated in Table 1, VOI analyses revealed significant correlations in the female corpus callosum and therefore confirmed the previously described TBSS results. Interestingly, FA values were negatively correlated with S100B, whereas regional radial diffusivity was positively correlated with serum S100B. In men, we did not find any significant correlation with S100B and FA, radial or axial diffusivity measures using this regional approach. Furthermore, a permutation test with 100,000 permutations regarding S100B serum levels and FA/radial diffusivity revealed significant gender differences in women compared to men (Table 1).

**Table 1:** Correlation values and permutation results for S100B, fractional anisotropy, radial and axial diffusivity in the corpus callosum.

sex	*p*-value	correlationcoefficient	significance of differencebetween correlation coefficients
**Fractional Anisotropy**			
female	**0.01**	**−0.59**	*p*-value
male	0.26	0.3	**0.004**
**Axial diffusivity** ( 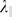 ).			
female	0.91	−0.03	*p*-value
male	0.7	0.11	0.68
**Radial diffusivity** ( 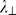 )			
female	**0.01**	**0.61**	*p*-value
male	0.22	−0.31	**0.002**

Note: Significant effects are written in bold.

The same result, which was obtained by computing the interaction effect of females and males regarding S100B levels, can be seen on the right side of Figure 1. Here, we show a significant lower FA (upper row, right) in the anterior parts (rostrum, genu) and body of the corpus callosum, parts of the superior longitudinal fasciculus and anterior and posterior forceps, and a significantly higher radial diffusivity (lower row, right) in the genu, rostrum and body of the corpus callosum and the anterior forceps in females compared with males. In addition to the FA results, we show significant results analyzing radial diffusivity in regions of the left external capsula and left uncinate fasciculus.

### Combining Serum Markers and T1-weighted Magnetic Resonance Imaging

**Figure 2:**
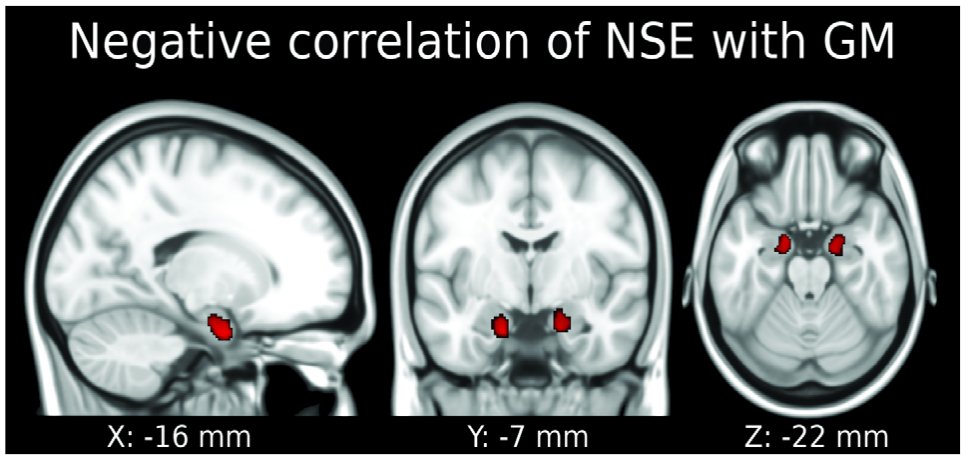
Gray matter density (GM) in both amygdalae and both most anterior hippocampi is correlated with serum neuron-specific enolase (NSE) in the female brain. No significant results were obtained for male subjects. *p*<0.05 family wise error was applied, reported coordinates are MNI coordinates. Neurological convention (left side of the brain is presented on the left).

Both S100B and NSE as glial/neuronal serum markers were analyzed for their explanatory power for T1-weighted MRI data, namely GM intensity. For S100B, we did not obtain any significant results with the chosen statistical threshold. In contrast, serum levels of NSE were correlated with regional GM density particularly in female subjects. Results of the VBM analysis are illustrated in Figure 2. We report data using a voxel-wise threshold of *p*<0.001 and show clusters which remained significant after correction using a cluster-wise threshold of *p*<0.05 family wise error (FWE). In female subjects, serum NSE was negatively correlated with GM density in the amygdalae and most anterior hippocampi bilaterally. No significant effects were obtained for male subjects with the chosen statistical threshold.

In a second step, we aimed to confirm this result and compare female and male groups in a regional analysis focusing onto the amygdalae. We computed a VOI analysis using the TD-ICBM Human Atlas in SPM and bilaterally selected the amygdaloid structures. These structures were used as masks for both, female and male groups, and smoothed GM values were extracted. Correlation of NSE and smoothed mean GM values, again corrected for covariates, were computed for both groups separately and can be seen in Table 2. Interestingly, both groups showed a significant negative correlation of GM density with NSE in the amygdaloid region in this VOI analysis – in contrast to the first whole-brain VBM analysis, where only females showed this association. Nonetheless, we failed to find significant gender differences using a permutation test with 100,000 permutations. We did not find any other significant correlation, difference or interaction of NSE and GM in any other group or combination of groups.

**Table 2:** Correlation values and permutation results for serum neuron-specific enolase and averaged smoothed gray matter density in the amygdalae.

sex	*p*-value	correlationcoefficient	significance of difference betweencorrelation coefficients
female	**0.0040**	**−0.66**	*p*-value
male	**0.0097**	**−0.62**	0.5

Note: Significant effects are written in bold.

### Histological and Whole Brain Gene Expression Analyses

**Figure 3:**
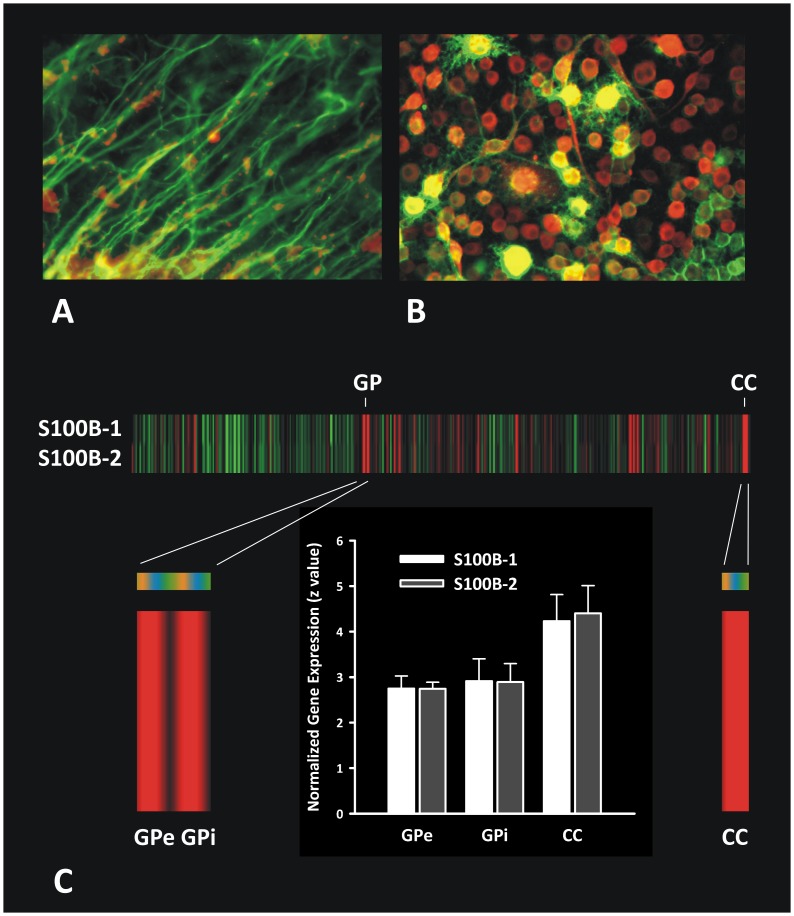
Expression and localization of S100B in the brain. (A) Co-localization (yellow) of S100B (red) and myelin basic protein (MBP)-positive (green) myelinated fibres in the human corpus callosum. Data originate from the subject previously published [6]. (B) Co-localization (yellow) between S100B (red) and the oligodendroglial marker p75 neurotrophin receptor (green) in the oligodendrocyte cell line OLN-93. (C) Individual normalized gene expression of S100B in heat map in z scores normalized to whole human brain expression, where green indicates relatively low and red relatively high expression. Subjects are coded in orange, blue and green (H351.2001,.2002 and.1009). Highest expression was detected in the corpus callosum (CC), followed by globus pallidus (GP). Bar chart shows quantitative values in CC and external/internal (e/i) GP (mean+standard deviation). Gene expression of S100B was analyzed in two probes: Probe 1 A_23_P143526; Sequence: AGCTTGATTTGCTTTGTGATTGAAAAATTGAAAACCTCTTTCCAAAGGCTGTTTTAACGG; Probe 2 CUST_17042_PI416261804; Sequence: AAGCTGAAGAAATCCGAACTGAAGGAGCTCATCAACAATGAGCTTT CCCATTTCTTAGAG.

In the histological study we examined co-localization between S100B and oligodendrocyte specific antigens in the human corpus callosum and cell culture by double immunofluorescence labeling. Figure 3 shows a close co-localization between S100B and oligodendrocyte specific myelin basic protein-positive myelinated fibres in the human corpus callosum, and between S100B and the oligodendrocyte/Schwann cell marker p75NTR in most oligodendroglial OLN-93 cells. Note that not all S100B positive cells are labelled as oligodendrocytes in Figure 3B as oligodendroglial markers are not expressed during all stages of oligodendrocyte development/maturation.

If we analyzed S100B gene expression in the whole human brain, S100B was most abundantly expressed in the corpus callosum, followed by the globus pallidus (Figure 3). The bar chart illustrates normalized z scores for these brain regions. All respective regions showed elevated S100B expression (Probe 1/2 corpus callosum *p* = 0.006, external globus pallidus *p*<0.05, internal globus pallidus *p* = 0.08/0.06; 2-tailed Student’s t test against 0), whereas there were no differences between probe 1 and 2 for each region warranting a high reliability (*p*>0.74; unpaired 2-tailed Student’s t test). Gene expression of S100B was, for both probes 1 and 2, higher in the corpus callosum than the external (*p* = 0.048/0.036) and internal globus pallidus (*p* = 0.08/0.057) without significant differences between both segments of the globus pallidus (*p*>0.6).

## Discussion

Our study aimed to validate the impact of serum markers on imaging data covering the GM and WM of the human brain. We focused on S100B because it is a glial protein that might act as a neuro- and gliotrophin inducing plasticity effects in the human brain [62]–[64].

As already stated in the introduction section we investigated the relationship between cell type specific serum markers and imaging parameters in a healthy cohort to exclude any impact of disease, because increased serum S100B may indicate glial alterations due to brain damage [65] in addition to functional secretion of S100B by astrocytes and/or oligodendrocytes [14]. Mathematical models suggest that serum S100B levels exceeding approximately 350 ng/l indicate brain damage [66]. Mathematical equations have been developed using values derived from literature and linearly fitting those values into a function in this study. Regarding neurological diseases with obvious brain damage, e.g. Creutzfeld-disease, mean expression levels are significantly increased to 395 ng/l [67], or in traumatic brain injuries with bad prognosis above 500 ng/l [68]. Accordingly, literature data supports the mathematically derived threshold. Lower levels below 300 ng/l have been detected in neuropsychiatric disorders, such as depression or schizophrenia and might describe a regenerative action through S100B, and due to their low concentration less likely a neurodegenerative process [8], [9]. Mean serum S100B was in the normal range of healthy subjects and far below this threshold in our subjects [45]. Likewise, normal NSE values exclude neuronal damage in our cohort [8], [54].

Our study shows that S100B is specifically related to WM structures in the healthy human brain as it correlated negatively with FA. We observed this effect in female subjects only, which might be related to higher serum levels of S100B, higher variance and a wider range of values of this glial protein in the female study group compared to male subjects. Furthermore, to the authors’ knowledge, there is no evidence to expect different expression pattern across sexes although our study shows significant correlations only within the female group. The results of the Allen Brain Atlas underpin the hypothesis of equal expression patterns across sexes, because all investigated subjects in this brain atlas are males, which show high S100B gene expression in the corpus callosum. Therefore, we conclude that due to the lower range of S100B values in our male cohort, we failed to show a significant correlation.

Our second more specific analysis revealed that correlations of serum S100B with FA in women had to be attributed to a positive correlation with radial diffusivity in the same regions without significant effects for axial diffusivity. For the GM we did not obtain any significant correlations with S100B. Remarkably, in these analyses, we corrected for potential influences of neuronal influences on DTI or VBM parameters by including the neuronal marker protein NSE as a covariate. For NSE, we did generally not detect any significant correlations with DTI parameters.

The close positive correlation of serum S100B with radial diffusivity fits well with our post mortem histological double immunofluorescence data demonstrating a high concentration of S100B in oligodendrocytes in the healthy human brain, particularly in the corpus callosum, the dorsolateral prefrontal, parietal and temporal WM (see results and Steiner et al. (2007) [6]). Most interestingly, the Allen Brain Atlas, which provides gene expression data across the whole human brain, indicates highest expression of S100B in the corpus callosum in agreement with our results and in the globus pallidus in agreement with the literature [69].

In sum and to our knowledge, our study is the first one validating the specificity of the glial protein S100B for brain changes in vivo. Our results support the assumption that radial diffusivity represents a myelin marker, whereas axial diffusivity represents an axonal marker, as already shown in [43], although this topic is controversially discussed [70]. Although, all subjects were healthy we detected negative correlations with FA, which can be interpreted as a loss of directedness, and positive correlations with radial diffusivity which might be coupled with a loss in myelinisation. One should note that the obtained serum markers are whole brain measures and for the first time correlated with imaging data. Our study suggests that in healthy subjects S100B is expressed in region wise patterns, which corresponds with high S100B expression in corpus callosum and globus pallidus. High S100B is associated with a reduction of FA and increased 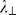
but does by no means support the idea of unhealthy or abnormal expression of S100B and/or diffusion parameters.

Beside DTI as WM parameter, both serum markers were analyzed in their explanatory power for T1-weighted MRI data by correlating them with GM density. Serum NSE was negatively correlated with regional GM density mainly in the amygdalae of both sexes, most pronounced in female subjects. One has to keep in mind that we controlled for serum S100B as a covariate in this analysis. We did not obtain any significant results for S100B in this analysis, although it is well known that human astrocytes contain and express S100B [6]. This might be related to a lower signal-to-noise ratio in the GM as it is composed of several other cell types beside astrocytes. In contrast, the close association between S100B and WM DTI parameters was observed mainly in the corpus callosum, where numerous fibers are oriented in the same direction yielding a very high signal-to-noise ratio [44]. Additionally, histological investigations showed highest concentration of oligodendrocytes in white matter in the corpus callosum, which might be an explanation for our sensitivity and the observed TBSS results [6]. This leads to the conclusion that S100B as an oligodendrocytic marker is closely related with radial diffusivity. An alternative view postulating a close relationship between S100B and astrocytes in the corpus callosum is very improbable, because 93% of S100B positive cells in the corpus callosum represent oligodendrocytes [6]. The same holds true for other structures in the white matter.

One might conclude that our study indeed investigated the ‘normal’ relationship between cell type specific serum markers and imaging parameters. Our results open a new perspective for future studies investigating major neuropsychiatric disorders – in particular major depression and schizophrenia, which have been discussed to be characterized by glial pathology [8], [47], [71] and affecting brain regions identified in our study [72], [73].
